# Supplementary material for: Uncovering community needs regarding violence against women and girls in southern Ethiopia: An explorative study
Source: PLoS One. 2024 Jun 11;19(6):e0304459. doi: 10.1371/journal.pone.0304459 (PMC11166345; doi:10.1371/journal.pone.0304459)
Supplement: S1 Annex — (DOCX) [file pone.0304459.s001.docx]

**Annex 6: Focus Group Discussion guides Information sheet** *To be modified depending the participants (girls/women and men-married unmarried)*

*Part I- Information Sheet*

**Study Investigators**:

Institute of Tropical Medicine, Antwerp, Belgium: Metasebia Admassu, Dr. Thérèse Delvaux, Professor Lenka Benova, Professor Marie Laga, Professor Koen Peeters Grietens and Dr. Claudia Nieto

Southern Nations Nationalities and Peoples Region Health Bureau, Hawassa, Ethiopia: Misganu Endrias

**Organizations:** Institute of Tropical Medicine, Southern Nation Nationalities and People’s Region Health Bureau

**Sponsor:** Institute of Tropical Medicine, Antwerp, Belgium

You are invited to participate voluntarily in a study conducted by Institute of Tropical Medicine in collaboration with Southern Nation Nationalities and People’s Region, health Bureau. Before you decide whether to participate or not in this study, it is important that you understand the information in this form, because it explains your rights and our responsibilities to you, the purpose, procedures, possible benefits, risks and inconveniences related to this study, and the right to refuse or stop your participation at any time.

Please, feel free to ask any questions at any time, for example about the possible benefits and possible risks related to this study. Your participation is completely voluntary. You can talk to anyone you trust about the research and you can take your time to think about whether you want to participate or not. In case you decide to participate, make sure you keep this background information and the signed consent form with you until the data collection is completed.

**Purpose and description of the study**

This study is being conducted to learn more about community perceptions of violence against women and girls and the challenges towards eliminating VAWG. The outputs of this study will be used to inform potential community-centered preventive and response activities by involving community members.

We believe that the findings from this study will be used to make recommendations for improvements in prevention and response practices towards the elimination VAWG in the local community.

We plan to collect information from community members, both men and women including girls, service providers, health extension workers, religious leaders, elders, police officers, and other important participants. We will do this through focus group discussions, interviews, informal conversations, and observations.

In this study, around 54-62 participants will take part from the community and two centers found in Arbaminch city.

**HOW THE STUDY IS DONE**

If you agree to be part of this study and if you meet the conditions to participate, you will be asked to respond to a set of questions that are related to violence against women and girls. But this does not mean that you are obliged to respond to all questions, you have the right to skip any questions which you do not want to give answer to. In addition to this, you can also fully withdraw from the study at any time during the data collection. As we said before, your participation in this study is entirely voluntary. Your decision to participate or to withdraw from the study is possible at any time.

All the information you provide during the focused group discussion is important for the study. Therefore, we will ask you to audio-tape the discussion, during audio-taping no names will be called up. The purpose of the audio-taping is to help the researcher to transcribe the discussion in order not to miss any content. The information on the recording will be deleted as soon as the transcription is done. In addition to this, be informed that the recordings will be accessed by the research team only. Should you still have any objections against audio-taping, you can discuss this with us and opt out. In this case, we will take notes during the interview. If you agree to participate in this study we will ask you to reserve one hour for the discussion.

**RISKS AND INCONVENIENCES**

The FGD will be conducted in private and comfortable place. During our discussion with you, there might be a chance that our questions will bring up some memories that happened in the past. If you need psychological support as a result of any disturbing memories, we will link you with support systems where you could get support from. This means you can meet us after the session privately or you can discontinue from the study at any time if you feel uncomfortable. Additionally, as we described above you have the right to skip sensitive questions. In case you need an assistant to go to seek legal service, health care, shelter or any other services, we will refer you to the place where you can get all the services you need.

**BENEFITS**

There are no direct personal benefits due to your agreement to participate in this study. However, your contribution will provide valuable insights to assist with the future prevention and response of violence against women and girls. The information collected from you may be used as a voice for future intervention in this community.

**COMPENSATION AND INSURANCE**

Any participation in a study involves a risk, however small it is. The organizer of this study, the Institute of Tropical Medicine, has obtained an insurance to cover any possible harm that may be caused to the participant directly indirectly caused by her/his participation in the study. If you get harmed or have questions about psychological trauma as a result of being in the study, please contact the researchers Metasebia Admassu and Misganu Endrias. They will be able to provide you with additional information concerning possible care.

**PRIVACY AND DATA PROTECTION**

We will do everything we can to protect your privacy. Information about you will be treated as strictly confidential; it will be stored in an electronic database, and identified by a code and not by your name. The documents where your name is mentioned will not be shared with anyone, except the study researchers, and few other people who have to keep it confidential, such as representatives of the Institute of Tropical Medicine.

We would like also to remind you that you are not allowed to share other participants’ information with others in the community.

All study data will be retained for a period of five years. The results of this study will be shared with you and those who participated in the research once the analysis is completed. We will disseminate the result through a study report, workshop and publication. Your data can be shared with other researchers for future research, only upon condition that they are pseudonymized (meaning that your name will be replaced by a code). Your name will not appear in any database, report or publication resulting from this study.

**ETHICS COMMITTEE**

Before the start, this study was reviewed and approved by the Ethics Committee of the institute of Tropical Medicine in Antwerp and local Ethics Committee from SNNP-RHB in Ethiopia.

**VOLUNTARY PARTICIPATION**

Your participation in this study is entirely voluntary. It is your choice whether you want to take part in it or not. Please note that you also have the right to stop your participation in the study at any time, even after you have signed this informed consent form. You do not have to give a reason for stopping. Also the researcher can decide stop your participation in this study at any moment as well, without asking your permission, if he/she judges this in your best interest, or if it appears that you do not follow the requirement for the participation. Should this happen, the researchers will explain this to you, and discuss what will happen afterwards.

**CONTACT PERSON IN CASE OF QUESTIONS**

If you have questions concerning your participation in this study, your right or if you think you have been harmed as a result of the study, please contact, now, during, or after the study:

Principal investigator

Metasebia Admassu

Email: [mjoffe@itg,.be](mailto:mjoffe@itg,.be)

Phone: +251911169187

Co-investigator (Ethiopia)

Misganu Endrias

Email: [Misganuendrias@yahoo.com](mailto:Misganuendrias@yahoo.com)

Phone : +251913020028

**Part II. Process, guidelines ground rules and participant information obtaining sheet– to be adressed after obtaining consent**

**Process and guidelines**

- We use codes or pseudonym during our discussions, you are not expected to tell your name
- A group facilitator will ask questions about your opinions, perception and experiences around VAWG in your area.
- The discussion will take up to 60 minutes and will be digitally audio recorded not to miss any information during the discussion.
- The researchers will also take note during the discussion.
- Your active participation in the discussion is encouraged, but you do not have to answer any questions you would prefer not to.

**Ground rules**

- It is recommended that one person talks at a time. There are no right or wrong ideas/answers. All ideas are equally important
- It is not advisable to judge other participants based on the idea they forward during the discussion

We ask participants to keep what is heard during the discussion confidential.

**Focus group discussion facilitator**:
**Co-facilitator** (if applicable):
**Geographic region**:

**# of Participants**:

Date: _______________________ Location: _______________________
Translation necessary for the interview: Yes No

Audio recorded: Yes No

If yes, the translation was from ____________________ (language) to ____ (language)

**Background Characteristics of Discussants**

Sex of FGD participants: Male Female

Marital Status Married Unmarried
Age of FGD participants:

15-20 years  21-26 years  27-32 years

33-38 years  39-49 years  >49 years

**Annex 7: FGD Semi-structured questions** *(Women, girls [married and unmarried], and Men [married and unmarried]*

**Organizations:** Institute of Tropical Medicine, Southern Nation Nationalities and People’s Region Health Bureau

**Study title**: **Uncovering community needs in reducing violence against women and girls in Southern part of Ethiopia: current practice and opportunities for change.**

1. **We would like to ask you a few questions about women and girls’ safety/security in this community**
   1. In this community is there a place where women and girls feel at ease or try to avoid? (day? night?) what makes this place insecure?
      1. Do they feel okay going to school at any time? How about the market, to fetch water or other places
   2. In this community if a woman or a girl has a security problem where do they go to seek assistance? Why?
   3. *Without mentioning names or indicating any one means*, according to you which group(s) of women and girls feel the most insecure or the most exposed risks of violence? Why? Which group(s) of women and girls feels the most secure? Why?
2. **We would like to ask you a few questions about how violence against women and girls (VAWG) is perceived in this community?**
   1. What do you think are the main forms of violence against children, adolescents, and adult women in your community?
   2. How do you understand VAWG/ How would you define VAWG?
   3. According to your understanding, is VAWG a problem in this community?
   4. What do you think are the causes of this violence?
   5. Have you heard a story about VAWG in this area? When? What happened? How did you hear about the information? What did you feel? *inform the participant not to mention any name while sharing the story*
   6. How often do you hear about VAWG in this community
   7. If a man/young person tells you about the violence he committed towards a girl or woman how do you react? Do you take it as good practice? Do you believe that men should beat or force their wives/partners or any girl in this community? Why? In what circumstances?
   8. Do you talk about VAWG in this area? **Probe**: What are the issues more talked about in this community?
   9. Are there any forms of VAWG accepted/taken as normal/ in this community? Which? Why
   10. According to you how does a family treat a woman or a girl who was the victim of rape or sexual assault? Why? How do they support her?
   11. What do women and girls do to protect themselves from violence? What does the community do to protect them? **Probe**: how?
   12. In your opinion what would be done in the community to create a safe environment for women and girls?
   13. What do you think should be done? By other bodies
3. **We would like to ask you some questions about different services available in your community towards prevention and response against VAWG**
   1. What do women usually do when they experience violence? Probe: physical violence? Sexual violence? Do they seek help ?
   2. Where do they go for seeking help? Why? Does she feel safe and comfortable to go to health facilities? Policy? Other formal sources?
   3. What services and supports are available for women and girls that are victims of violence? Probe: (counseling, women’s groups, legal aid, shelter, etc..)
   4. What additional services you need in the community?
4. **We would like to ask you a questions about a possible incidence: the below cases are contextual and culturally appropriate. The story will not have a name, it is not linked to anyone of the participant or any specific community. But it will help us to discuss based on scenario**
   1. Case study 1. A young girl 17 years old, goes to a school crossing a bush. While she was going to school she wanted to use a community latrine, the latrine is found away from the main road, she goes few meters from the main road and uses the latrine. When she goes out she faced unexpected incidence, a young man grabbed her, pulled her behind the latrines and raped her.
      1. If a women or a girls reported that she experienced violence similar to the girls in the story, how many of you would believe her story?
      2. Why do women and girls hesitate to share experiences like this with other people?
      3. Where could this women go to receive appropriate assistance? What kind of assistance and support could she receive?
      4. What other sources and services do you think should be in place
   2. Case story b. A married women was beaten severely by her husband following her argument with him. He was very angry because she was arguing with him. They had big fight, and he beat her, following the physical violence, the same night he raped her without her willingness to have sex with him. She was angry at him and left the house. /*Ask the above questions*/
5. **We would like to ask you about the challenges and possible solutions to work towards the elimination of VAWGs in your community**
   1. According to you, what are the biggest obstacle to make your community free from violence? Do you think this can be prevented? How? Who do you think is responsible? what things should be done?
   2. What else do you have?

**Conclusion**

At the end of the discussion we thank the participant for their time and their contribution. We also remind the participants the purpose of the discussion. We will also explain the next step and repeat what we will do with the information and what purpose it will eventually serve. We will also remind participants not to share information or the names of other participants with others in the community. Finally we will ask the participants if they have question. If anyone wishes to speak in private, respond that the facilitators will be available after the meeting.

**Annex 8: In-depth interview with Violence survivors** *(Women, girls and minors)*

**Organizations:** Institute of Tropical Medicine, Southern Nation Nationalities and People’s Region Health Bureau

**Study title**: **Uncovering community needs in reducing violence against women and girls in Southern part of Ethiopia: current practice and opportunities for change.**

| *Participant ID #* |  |
| --- | --- |
| *Age* |  |
| *Marital status* |  |
| *Scholing/employment* |  |
| *Interview date and time* |  |

**Can you please tell me a little about yourself?**

a. How do you normally spend your day?

b. Do you go to school? What grade are you? Do you have a job? What do you do?

c. What things do you like to do

d. How old are you?

1. **We would like to ask you about the safety and security of women and girls in this community**
   1. What are the situations that pre-dispose women and girls to different forms of violence?
   2. What forms of VAWG do you think occur the most in this community? Why?
   3. Who are the most at-risk population group? Why?
   4. When and where does VAWG occur in this community?
   5. Who are usually the perpetrators of VAW in this community?
   6. What usually happens to perpetrators if they are caught?
   7. Has the problem of VAWG in this community got worse? Better? Or stayed the same? (**probe** for particular types of violence that have gotten worse, better, or stayed the same. If there has been a change, probe for what has caused it?
2. **We would like to ask you about the Perception and help-seeking behavior of VAWG in this community**
   - - 1. Where do you go first to seek assistance? Probe: Why did you choose to go there? Who told you to go there? Who do you see there? What did they say to you? How did they treat you? How did you feel? What helped you? What didn’t? what was difficult? Are you satisfied with the service you get? Why/why not? Did anyone go with you? What steps did you take to seek care after the attack?
       2. How long did it take between the assault and the recourse?
       3. How did you know about the service? Who made the decision to use these services? How did you decide?
       4. What did you expect from the service renders? Did you find the service helpful? In what way? Who helped you to go through this process?
       5. Did you go elsewhere afterward? Where? Whom do you contact? Why did you go there?
       6. In your opinion where do other girls/women go when they experience violence? **Probe**: why? Do they go to a health facility? Why? Why not? Do they go-to legal service? Why? Why not? Do they go to the woman/gender office? Why/why not?
       7. Are there any types of VAWG accepted in this community? Which? Why?
       8. What barriers do women and girls face in seeking care? (**probe**: for example, stigma against survivors, lack of coordination between services, lack of follow-up, lack of service?)
3. **We would like to ask you how you are feeling today and if it is possible to talk about the issue of VAWG**

- What were the consequences for you of the VAWG you suffered from? (physical, psychological, social, educational, economic) for the victim? -> It is important to understand how these consequences are linked to each other and to grasp the real effects, using concrete examples.
- What were the consequences for those close to the victim?
- What were the reactions of relatives and the community?

-Towards her? Towards the perpetrator?

- What does she think explains such reactions?
- What was the impact of these reactions on the victim, and how did she experience them

**IV. Recommendation for reducing/eliminating VAWG in the community**

- 1. What does the community do to prevent VAWG in this community?
  2. What is currently being done in this community to prevent VAWG?
  3. What can elders do to prevent VAWG in this community?
  4. What can HEWs do to prevent VAWG in this community?
  5. What can religious leaders do to prevent VAWG in this community?

1. **What can other members of the community do to prevent VAWG in this community? Recommendation for improving VAWG services**
   1. What does the community do to respond to VAWG in this locality?
   2. What do you think needs to be done to break the barriers women and girls face to reporting VAWG? **Probe**: what else
   3. What do you think needs to be done to break the barriers women and girls face to access post-violence services in the community? **Probe**: what else
   4. What services need to be available in this community to help address VAWG?
   5. What should be done to help VAWG survivors in this community? (**Probe**: how could these efforts be improved?)
2. **Recovery/follow-up care**
   1. How do you feel now? Do you feel recovered from the violence trauma? What makes you think so?
   2. What helped you to get better? How long did it take?
   3. What remains difficult for you?
   4. What are the missing services? What do you wish to be improved?

Is there anything you would like to mention?

Thank you for your time!!

**Annex 9: Interview guide for key informants** (*Police, HEWs, religious leaders, elder, gender officer)*

**Organizations:** Institute of Tropical Medicine, Southern Nation Nationalities and People’s Region Health Bureau

**Study title**: **Uncovering community needs in reducing violence against women and girls in Southern part of Ethiopia: current practice and opportunities for change.**

| Participant ID |  |
| --- | --- |
| Organization |  |
| Position in the community |  |
| Current profession |  |
| Sex |  |
| Marital status |  |
| Interview date and time |  |

1. **Background information**

Can you tell me a little about yourself? **Probe** the level of education and employment

Age:

Married/Unmarried/Divorced

Number of children

1. **Survivors and community Perception of VAWG**
2. How do you understand VAWG?

How do other people in this community understand VAWG?

1. What is your experience around VAWG
   1. Have you heard a story about VAWG in this area? **Probe**: When? What happened? How did you hear about the information? What did you feel?
   2. How often do you hear about VAWG in this community?
   3. In your area where do women/girls go when they experience violence? **Probe**: why did they choose the place?
   4. Whom do they trust more? Why
   5. Do you talk about VAWG in this area? **Probe**: What are the issues talked about in this community?
   6. What would you do if you witness a woman/girl beaten by her husband/partner? **Probe**:
   7. Are there any forms of VAWG accepted in this community? Which? Why?
   8. In your opinion, is violence decreasing or increasing? **Probe**: Why? How? Why do you think VAWG is common in this community?
2. **Understand major challenges towards eliminating VAWG in the community**
   1. What preventive activities are given in the facility you are working?
   2. In your opinion, do you think VAWG can be prevented? **Probe**: why? how?
   3. In your opinion what are the challenges towards eliminating VAWG in the community? **Probe**: what else?
3. **Exploring current community-led preventive and response practices toward VAWG**
   1. What response activities are provided at your level? **Probe**: what else?
   2. Are there practices or interventions in the community towards the prevention of VAWG? How about response practices? **Probe**: can you tell me a little bit about it? How did you hear about it? How often do you get the information?
   3. In your opinion do you think a woman/girl should seek help when she faces violence? **Probe**: why? Why not?
   4. In this community when a woman/girl faces violence, where do they normally go? **Probe**: why there?
   5. How do you collaborate with different stakeholders working around VAWG
4. **Identifying additional sustainable community-led preventive and response activities**
   1. In your opinion do you think there are enough practices in the community to prevent VAWG? How about the response to survivors of violence?
   2. What additional practices do you think should be in place to eliminate VAWG in this community? **Probe**: who do you think should be responsible to prevent violence? Why? How?
   3. What additional response activities do you think should be in place for survivors of violence? **Probe**: who should be responsible to respond? Why? How?

Is there anything you would like to mention?

Thank you for your time and insight!!

**Annex 10: Interview guide for Service providers at the one stop-center and safe house**

**Organizations:** Institute of Tropical Medicine, Southern Nation Nationalities and People’s Region Health Bureau

**Study title**: **Uncovering community needs in reducing violence against women and girls in Southern part of Ethiopia: current practice and opportunities for change.**

| Participant ID |  |
| --- | --- |
| Organization |  |
| Position |  |
| Current profession |  |
| Previous role in health facilities (Y/N) |  |
| Service year in current position |  |
| Sex |  |
| Interview date and time |  |

1. **Background information**
   1. Tell me about your time at this facility. How long have you worked here? What is your current role? What was your previous role in this facility/if any/?
   2. What type of victims do you work with?
   3. What does your work consists of? How does this vary? For what reason?
2. **Understanding and perception of VAWG**
   1. How do you understand or define VAWG?
   2. Other than those coming to this facility, have you come across a service provider who is a victim of violence? When was that? How did you feel about it? How did the provider deal with the situation?
   3. According to your understanding is violence a problem in this community? Why? How so?
   4. Have you received specific training on VAWG? **Probe**: how long was the duration of the training? Who provided the training? Did you receive any relevant training as part of your formal education? What is it on?
   5. What types of VAWG are usually reported at this facility? (**Probe** for a specific type: sexual, physical, psychological..)
3. **Facility response towards recovery**
   1. What care or interventions do the victims believe play a role in the physiological recovery of victims? How about their reintegration into the community?
   2. What care or interventions do the providers believe hinder or prevent the victim's recovery?
   3. According to the profession, which ones do victims most commonly turn to?
   4. For which situation do victims use or not use care, assistance, and support services?
      1. What are the socio-cultural characteristics of those who do use them?
   5. What are the main socio-cultural barriers to victims’ use of such services, organizations, and institutions?
   6. What is the role of family and community members in the process of recovery and socio-economic reintegration of victims?
   7. Imagine that someone visits this facility. How do you determine whether she is a survivor of violence? How does the community know about the response activity towards violence in the facility? How do you reach them?
   8. What are the specific individual symptoms and sufferings that victims exhibit? What approaches are used?
   9. Anything you do to reach the perpetrator? How? What happens? What are the mechanisms
   10. Which other levels of the health system provide VAWG response services? What other institutions other than the health system?
   11. What motivates community members to access this facility for VAWG prevention and response services?
   12. In what way do you interact with VAWG survivors in your work?
   13. How do you record and track VAWG cases at this facility? How is the information used? (**Probe**: for the existence of registration book, reporting mechanism, how the facility uses the data for decision making and for making changes)
   14. How does this facility ensure confidentiality of VAWG survivors’ information and records?
   15. What additional training or knowledge would help you and your colleagues to support VAWG survivors who visit this facility?
   16. What policies/guidelines/protocols are in place in this facility to work with VAWG survivors.
4. **Preventive activities toward VAWG**
   1. What types of VAWG prevention services currently exist in this facility? Where are these services found within the facility (**Probe**: which department, how do you coordinate?),
   2. Who currently provides these preventive services? What resources do you have?
   3. What do healthcare workers do to prevent VAWG in the community? How about HEWs or volunteers do?
   4. Think about any intervention that has contributed to the reduction of VAWG in this community? What are the preventive activities conducted? What impact did it bring?
5. **Recommendations for the prevention and response of VAWG**
   1. What other type of support or resources would help to ensure the prevention and responses towards the elimination of VAWG in this community?
   2. Is there anything else you want to tell us, because we didn’t ask the right question? What question do you have for us?

Thank you very much for your time and insights. They are very much helpful.

**Annex 11: Site Observation Guide**

**Organizations:** Institute of Tropical Medicine, Southern Nation Nationalities and People’s Region Health Bureau

**Study title**: **Uncovering community needs in reducing violence against women and girls in Southern part of Ethiopia: current practice and opportunities for change.**

Facility ID Number: ____________________

Study-generated ID for the observation: ____________________

Time observation is initiated:

**Instructions**

While conducting the observations please take note of everything you see regarding the setting, client flow, infrastructure, privacy and confidentiality, and posters. Collect the information guided by the below questions but not limited to!

- - - 1. Where is the facility? ( stand-alone, integrated), is there a signpost, any indication of physical accessibility
      2. Registration book, reporting forms,
      3. Is there a place dedicated to providing counseling sessions for violence survivors
      4. Can conversation within the examining room be heard outside of the room?
      5. Can the client be seen from outside the examining room?
      6. Are there interruptions during the consultations?
      7. Are there national guidelines in place
      8. Are there educational material (e.g. posters, leaflets, job aids)

Additional Note:

**Annex 12. Semi-Structured question guide for key informant interview:** *(one-stop center and safe house manager)*

**Organizations:** Institute of Tropical Medicine, Southern Nation Nationalities and People’s Region Health Bureau

**Study title**: **Uncovering community needs in reducing violence against women and girls in Southern part of Ethiopia: current practice and opportunities for change.**

| Participant ID |  |
| --- | --- |
| Organization |  |
| Current profession |  |
| Previous role in the facilities (Y/N) |  |
| Service year in current position |  |
| Sex |  |
| Interview date and time |  |

1. Tell me a little about yourself? Your educational background? Position? Year of service in the current position? Your previous role?

2. What response services do you provide at the safehouse/one-stop center for VAWG survivors?

3. What preventive services do you provide at the facility against VAWG?

4. Who operates this facility? NGO /local international/, government …

5. How does the community know about the service availability at this facility?

6. How do you see the number of violence cases in this facility? Is it increasing? Decreasing? How do you use the data? Is there a regular meeting with the data managers?

7. What are the challenges in addressing VAWG in the facility? What are the gaps? What else?

8. How is the follow-up mechanism to the service? To the client?

9. How do you collaborate with other sectors? With which institution or organization is the collaboration?

- How does this collaboration take place?
- What is the basis for this collaboration? What are the experiences of collaboration? Do you have regular meeting? What are the changes or actions you take after the meeting/*ask a specific scenario*/
- What are the obstacles and/or facilitators to cooperation?
- What needs are not yet covered by existing services in the region? What is missing in the circuit today?
- How should the multi-sectoral care circuit for victims be organized?
- How to ensure the quality of integrated care?

10. What needs are not yet covered by existing services in the region? What is missing in the circuit today?

- How should the multi-sectoral care circuit for victims be organized in the zone?
- How to ensure the quality of integrated care?
