# Supplementary material for: Uncovering community needs regarding violence against women and girls in southern Ethiopia: An explorative study
Source: PLoS One. 2024 Jun 11;19(6):e0304459. doi: 10.1371/journal.pone.0304459 (PMC11166345; doi:10.1371/journal.pone.0304459)
Supplement: S1 File — (DOC) [file pone.0304459.s002.doc]

Table-S2. Description of key thematic areas, subtheme, and codes (VAWG in Ethiopia, August 2022, Arbaminch City Ethiopia)

| **Key thematic categories** | **Sub-themes** | **Codes** |
| --- | --- | --- |
| Perception and understanding of VAWG | Human right | Young girls |
|  | A minor, rare phenomenon, no VAWG | Normalization |
|  | Married women should expect VAWG | Normalization |
|  | Tolerated (acceptable violence) | Violence to a sex worker, violence within a marriage [without major wound], violence to a girl who dresses inappropriately, verbal abuse, violence that occurs in a risky area and hour, attempted rape |
|  | Unacceptable violence | Violence against a child and shy/calm girl, Losing virginity following sexual violence, Availability of confirmatory signs following the violence, and repeated violence |
| Community and female violence survivors’ responses to the act of VAWG | Community response to the act of VAWG | blame, non-interference, stigma, discrimination, gossip, feeling pity, tolerance towards the perpetrator, bringing to the legal, customary mediation compensation, marrying the rapist, hiding evidence, supporting survivors |
|  | Family response to the act of VAWG | Family honor, Seeking legal service, seeking compensation |
|  | Survivors’ response to the act of VAWG | Silence, non-disclosure, tolerance, separation, leaving the neighborhood/hometown, and taking action |
| Existing services, access, and service delivery barriers toward VAWG | Existing services | The community-based, school-based, facility-based |
|  | Care seeking experience | Shame, fear,… |
|  | Barriers to care seeking /access/ | The community-based, School-based interventions, and facility-based |
|  | Barriers to service delivery | Integration, collaboration, lack of resource |
| Perceived contributor factors towards VAWG | Individual level | Age, sex, lack of knowledge, low economic status, addiction |
|  | Relationship level | Poor communication, instability within the family |
|  | Community level | Safety and security, |
|  | Societal level | Gender inequality, poverty, |
| Suggested response and preventive activities toward VAWG | Individual level | Disclosure, self-care, individual support |
|  | Relationship level | Mother-to-daughter communication, partner communication |
|  | Community level | Emotional support, avoiding customary mediation, condemning the act of violence |
|  | Societal level | Justice, continuous support, economic empowerment, youth-related services, school-based programs |
