# Supplementary material for: Uncovering community needs regarding violence against women and girls in southern Ethiopia: An explorative study
Source: PLoS One. 2024 Jun 11;19(6):e0304459. doi: 10.1371/journal.pone.0304459 (PMC11166345; doi:10.1371/journal.pone.0304459)
Supplement: S3 File — (DOC) [file pone.0304459.s004.doc]

**Table-S4. List of existing services in Arbaminch city by the time of data collection as described by study participants.**

|  | **Women and girls** | **All study participants** | **Violence survivors** | **GBV service providers and implementers** | **Remark** |
| --- | --- | --- | --- | --- | --- |
| **Prevention activities on GBV** | | | | | |
| Awareness creation through a radio program in the local language |  |  |  | **🗸** |  |
| School-based awareness creation (parliament and gender clubs) | **🗸** |  |  | **🗸** | Reported by two young girls who are participating in the program |
| Empowering girls through school-club | **🗸** |  |  |  | Reported by two young girls who are participating in the program |
| Community conversation |  |  |  | **🗸** |  |
| Village-level armed men | **🗸** |  |  |  |  |
| **Response services** | | | | |  |
| One-stop center (Medical and legal support) | **🗸** |  |  | **🗸** |  |
| Safehouse (Temporary shelter and follow-up on the legal decision) |  |  | **🗸** | **🗸** |  |
| Women and Children’s affair office |  | **🗸** |  |  |  |
| Police, and free legal service |  | **🗸** |  |  |  |
| Customary mediation |  | **🗸** |  |  |  |
