# Supplementary material for: Uncovering community needs regarding violence against women and girls in southern Ethiopia: An explorative study
Source: PLoS One. 2024 Jun 11;19(6):e0304459. doi: 10.1371/journal.pone.0304459 (PMC11166345; doi:10.1371/journal.pone.0304459)
Supplement: Attachment — Submitted filename: 1603 - approval letter amendm.pdf [file pone.0304459.s005.pdf]

Institutional Review Board

Mrs. Metasebia Admassu Joffe
Department of Public Health

IRB/RR/AC/132	OUR REF.	ATTACHMENT(S)	ANTWERP
	1603/22		19/07/2022

Concerns: Amendm 1: Uncovering community needs in reducing violence against women and girls in Southern Ethiopia: current practice and opportunities for change; v. 3.0, dd July 2022

I am pleased to inform you that after review by the IRB Chairperson, the above mentioned amendment has been approved on July 7th, 2022.

We wish to remind you of the following important aspects:

- It is the researchers' responsibility to secure ethics approval in the study country(ies) as required by local regulations, before any study-related activities are started.
- The IRB (+ EC UZA when applicable) should receive a yearly update report to the IRB at the latest one year after the approval date, and an end-of-study report.
- According to the Belgium biobank law, all human material (blood, urine, etc) that is used for research must be registered in a biobank. The sample-related data should be recorded in the ITM biobank database. If research samples are transiting or stored at ITM, this 'Biobank Law' applies. Please contact the Biobank manager via biobank@itg.be.
- In case you have any questions about data protection or the GDPR, please contact ITMs Data Protection Officer via informatieveiligheid@itg.be.
- Research studies prospectively involving human participants should be registered in one of the WHO-accepted primary registers (e.g. www.clinicaltrials.gov).

Kind regards,

Dr. Raffaella Ravinetto

Chairperson Institutional Review Board
